# Correspondence on impact of COVID-19 on O&G trainees

**DOI:** 10.52054/FVVO.14.2.021

**Published:** 2022-07-01

**Authors:** P Sookaromdee, V Wiwanitkit

Dear Editor,

We would like to correspond and share ideas on the publication “The impact of COVID-19 on O&G trainees; where are we now? (Duggan et al., 2022).” Despite the “establishment” of normal services, the deleterious impact on trainees, particularly in benign gynecology surgery training, remains, according to Duggan et al., (2022). Addressing pre-pandemic training shortages while also addressing the surgical backlog and service provision needs may take years (Duggan et al., 2022). We agree that there has been a disruption of normal services and training pathways during the COVID-19 pandemic. In many previous reports, it has been claimed that tele-education could be an effective tool for supporting training/education during the pandemic and learners may be satisfied with this technology. Furthermore, some learners also suggested that tele-education should form part of standard training post pandemic (Armon et al., 2021). This current report suggests, after resuming normal practice, tele-education cannot actually replace standard training and satisfaction cannot guarantee the outcome proficiency of the learner. At present, the urgent requirement is establishing additional training sessions for the learner to regain and improve practical skill for further professional healthcare development.
